# Cancer screening among racial/ethnic groups in health centers

**DOI:** 10.1186/s12939-020-1153-5

**Published:** 2020-03-27

**Authors:** De-Chih Lee, Hailun Liang, Nanqian Chen, Leiyu Shi, Ying Liu

**Affiliations:** 1 Department of Information Management, Da-Yeh University, No.168, University Rd., Dacun, Changhua, 51591 Taiwan, R.O.C.; 2 Johns Hopkins Primary Care Policy Center, Johns Hopkins Bloomberg School of Public Health, Baltimore, MD 21205 USA; 3 Renmin University of China, School of Public Administration and Policy, Beijing, China

**Keywords:** Cancer prevention, Racial disparities, Health center

## Abstract

**Background:**

Underserved and low-income population are placed at a disadvantage for receiving necessary cancer screenings. This study aims to measure the rates of receiving three types of cancer screening services, Pap test, mammogram and colorectal cancer screening, among patients seen at U.S. health centers (HCs) to investigate if cancer screening among patients varies by race/ethnicity.

**Methods:**

We analyzed data from the 2014 U.S. Health Center Patient Survey, and included samples age 21 and above. We examined three cancer screening indicators as our dependent variables including cervical, breast, and colorectal cancer screening. Logistic regressions were used to assess the racial/ethnic disparities on cancer screening, while controlling for potentially confounding factors.

**Results:**

The rates of receiving three types of cancer screening were comparable and even higher among HC patients than those for the U.S. general population. Both bivariate and multivariate results showed there were racial/ethnic differences in the likelihood of receiving cancer screening services. However, the differences did not favor non-Hispanic Whites. African Americans had higher odds than Whites (OR: 1.92, 95% CI: 1.44–2.55, *p* < 0.001) of receiving Pap tests. Similar results were also found in measures of the receipt of mammogram (OR = 1.96, 95% CI: 1.46–2.64, *P* < 0.001) and colorectal cancer screening (OR = 1.28, 95% CI: 1.02–1.60, *p* < 0.05).

**Conclusion:**

The current study presents U.S. nationally representative estimates and imply that HCs are helping fulfill an important role as a health care safety-net in reducing racial/ethnic disparities in the delivery of cancer screening services.

## Introduction

Cancer is the second leading cause of death in the world and was responsible for an estimated 9.6 million deaths in 2018. Globally, about one in six deaths is due to cancer. Lung, prostate, colorectal, stomach and liver cancer are the most common types of cancer in men, while breast, colorectal, cervix, lung, and thyroid cancer are the most common among women [29]. Cancer mortality can be reduced if cases are detected and treated early. Between 30 to 50% of cancer can be prevented by implementing evidence-based preventive strategies and addressing risk factors [29]. Breast cancer is the second most common cancer among women in the United States. According to the United States Preventive Services Task Force (USPSTF), women who are 50 to 74 years old and are at average risk for breast cancer should get a mammogram every 2 years [25]. The USPSTF also recommends screening for cervical cancer in women age 21 to 65 with a Pap smear every 3 years [26]. However, the rates of cervical cancer in the U.S. have gone down in recent years [26]. In terms of colorectal cancer, a common cancer for both men and women in the United States, the USPSTF recommends screening using fecal occult blood testing every year, sigmoidoscopy or colonoscopy ever 10 years, beginning at age 50 and continuing until age 75 [27].

According to the 2010 U.S. Census, approximately 36% of the population identified themselves as belonging to a racial or ethnic minority group [28]. Though health indicators such as infant mortality and life expectancy have improved for most Americans, disparities in health care and health outcomes persist for certain racial/ethnic groups [20]. Because of social, economic, and/or environmental disadvantage, racial and ethnic minority groups may face barriers regarding access to care and achieving good health outcomes [3, 11, 14, 19]. Underuse of cancer screening services may result in delayed diagnosis, missed treatment opportunities, and poorer survival and outcomes [6, 13]. Over the years, efforts to reduce disparities and achieve health equity have been made. The federally qualified health centers, also referred to as health centers (HCs), have been providing affordable and quality primary care for medically underserved and vulnerable populations including racial/ethnic minorities since 1960s [9]. HCs provide culturally competent care, such as language translation services to non-English speaking patients, to build clinician-patient trust and reflect the racial diversity of its patients [7]. In 2014, 1278 HCs provided care to 22.9 million Americans; among them, around 58% were racial/ethnic minorities [7].

The statistics related to cancer screening vary by race/ethnicity in many studies. A study focused on racial and ethnic disparities in colorectal cancer screening found that African American, Hispanic, and other racial/ethnic groups were less likely than non-Hispanic Whites to receive a colorectal endoscopy [23]. Racial disparities have also been documented in breast and cervical cancer screening among African American, Hispanic, and Asian populations, and perceived discrimination may contribute to this issue [12]. One study showed that non-Hispanic Whites with lower socioeconomic status also faced health disparities in cancer prevention, which may be due to shared health risks with their poor minority urban counterparts for not obtaining preventive care [4].

While previous studies have examined the association between racial/ethnic groups and cancer screening among the general population, few studies have provided evidence among health center patients. Study of racial and ethnic disparites among health center patients is important because in the U.S. health centers are largely funded by the government and serve as a safety-net provider for vulnerable populations including racial and ethnic minorities. The mission of the health center program is to reduce and eventually eliminate disparities in access to and quality of care across subpopulations within the U.S [10].

This study aims to measure the rates of receiving three types of cancer screening services among patients seen at HCs to investigate if cancer screening among patients varies by race/ethnicity, and to test if cancer prevention provided by HCs could mitigate health disparities caused by racial/ethnic differences. We used data from the 2014 Health Center Patient Survey, which was the latest and most nationally representative survey of U.S. HC patients. Differences between the racial/ethnic groups of HC patients can help shed light on the implications of the role of HCs in providing a usual source of care to all patients and in promoting health care equity.

## Methods

### Data sources

We analyzed data from the 2014 Health Center Patient Survey. The Health Center Patient Survey is a person-administrated survey of Health Center Program patients. It is unique in its focus on comprehensive patient-level data and its design to provide a nationally representative view of patients served by grantees under Section 330 of the Public Health Service Act. It was conducted in 1998, 2002, 2009, and 2014, developed by the U.S. government Health Resources and Services Administration under the Department of Health and Human Services. The Patient Survey implemented a three-stage sampling design. First, 169 HC grantees were recruited, then 520 HC sites were contacted operating within those participating grantees, and lastly, 7002 patient interviews were conducted. The 2014 Health Center Patient Survey had a probability sample of 7002 patients representing over 22 million patients seen at HCs during 2014. We included samples age 21 and above. The final sample size of the study was 5453.

### Measures

In this study, we examined three cancer screening indicators as our dependent variables, which represent commonly used measures of preventive care utilization. The age to begin screening is in accordance with the standard for health insurance coverage under the Affordable Care Act (or commonly called Obama Care). All these measures were coded as dichotomous variables (“recent screening = 1” or “no screening = 0”). The three preventive measures included (a) receiving a Pap test in the past 3 years among women age 21 to 70, (b) receiving a mammogram in the past 2 years among women age 40 and older, and (c) receiving a colonoscopy/sigmoidoscopy in the past 10 years or fecal occult blood test in the past year among adults age 50 and older. The main independent variables of interest were race/ethnicity categories, including non-Hispanic White, Hispanic or Latino, non-Hispanic African American, and others.

We used the access-to-care model of Aday et al. [1] to select covariates potentially associated with the outcome measures. The covariates were categorized as predisposing (including age, gender, education level, weight and tobacco use), enabling (including health insurance status and usual source of care) and need factors (including personal history of cancer). These variables were coded as follows: age (continuous), gender (male versus female), education level (below high school versus high school graduate), body mass index (BMI) (normal/overweight/obese), current smoker (yes versus no), currently insured (yes versus no), usual source of care (yes versus no), and personal history of cancer (yes versus no).

### Statistical analysis

Using a cross-sectional analysis of the data, we first described patient characteristics and receipt of preventive services across racial/ethnic groups of HC patients. Next, we used regression analyses to identify the associations between race/ethnicity and cancer screening, controlling for patients’ other sociodemographic and health-related characteristics. In each model, we used simple logistic regression to assess the racial/ethnic disparities on cancer screening, and additionally adjusted for potentially confounding factors including age, gender, education level, weight, current smoker/cigarettes, currently insured, usual source of care, and personal history of cancer. The 2014 Health Center Patient Survey was based on a complex survey design, which applied a three-stage sampling design (HC grantees – HC sites - patients) to reflect a nesting structure. Using Stata/SE version 14.0, statistical analyses were performed while accounting for the complex sampling design. Two-tailed *P* values less than or equal to 0.05 were considered statistically significant.

## Results

### Sociodemographic, health-related characteristics and receipt of cancer screening by patients at HCs

Table 1 describes the patient characteristics and receipt of three types of cancer screening services across racial/ethnic groups. We included samples aged 21 and above. The final sample size of this analysis was 5453, representing around 17.5 million patients seen at HCs during 2014. In terms of predisposing factors, the mean age of the respondents was 45.4 years. Weighted percentage showed a greater proportion of patients was female (63.4%). Non-Hispanic White and Hispanic/Latino patients represented 24.6 and 33.9% of the population, respectively, while non-Hispanic African Americans and non-Hispanic others accounted for 23.4 and 18.1%, respectively. About 66.2% of the sample had a high school education. Around 35.5% of Hispanic/Latino patients reported overweight, higher than the other three groups, while 57% of non-Hispanic White and 57.5% of non-Hispanic African American patients had obesity issues. Non-Hispanic White patients reported higher rates of current smoking (38.1% vs. 28.8% on average). Regarding enabling factors, only 60.0% of Hispanic/Latino respondents had health insurance, which was 12.9% lower than the mean. Most patients had a usual source of care. In terms of need related factors, more non-Hispanic White respondents had a personal history of cancer compared with the sample average (8.9% versus 6.3%).

**Table 1:** Cancer screening, predisposing, enabling, and need determinants of 2014 health center patients 21 years and older

	Total (*n* = 5453)	Non-Hispanic White (*n* = 1342)	Hispanic or Latino (*n* = 1847)	Non-Hispanic African American (*n* = 1278)	Non-Hispanic “other” (*n* = 986)	***P***-value
**Predisposing**						
Age: Mean (SE)	45.4 (0.51)	48.3 (0.76)	40.4 (0.87)	43.2 (0.96)	46.1 (2.12)	**< 0.001**
Female	63.4%	62.4%	64.5%	64.5%	64.7%	0.8895
High school graduate	66.2%	72.3%	49.5%	69.7%	67.4%	**< 0.001**
Overweight (25.0 < BMI < 29.99)	26.1%	24.3%	35.5%	21.2%	22.9%	**< 0.001**
Obese (30.0 < BMI)	52.9%	57.0%	44.4%	57.5%	33.5%	**< 0.001**
Current smoker (cigarettes)	28.8%	38.1%	12.2%	26.6%	19.8%	**< 0.001**
**Enabling**						
Currently insured	72.9%	75.5%	60.0%	77.8%	84.9%	**< 0.001**
Has a usual source of care	98.0%	97.5%	97.9%	99.4%	98.3%	0.2645
**Need**						
Personal history of cancer	6.3%	8.9%	3.3%	3.2%	6.1%	**0.0001**
**Cancer Screening**						
Had Pap test in past 3 years *Women 21–70 years*	81.9%	72.8%	92.2%	89.9%	87.2%	**< 0.001**
Had mammogram in past 2 years *Women 40 years and over*	70.3%	64.2%	76.4%	81.4%	80.3%	**0.0019**
Colonoscopy or sigmoidoscopy in past 10 years, or Fecal Occult Blood Test in past year *Men and women 50 years and over*	60.2%	58.6%	59.3%	61.3%	74.9%	0.2694

With respect to cancer screening, 81.9% of female HC patients aged 21 to 70 received Pap tests in the past 3 years, 70.3% of women age 40 and above had a mammogram in the past 2 years, and 60.2% of HC patients age 50 and above received a colonoscopy/sigmoidoscopy in the past 10 years or fecal occult blood test in the past year. Significant differences across racial groups were found in the receipt of Pap tests and mammograms. Compared with other racial/ethnic groups, non-Hispanic White HC patients generally had lower rates of recent screening. Specifically, more than 90% of Hispanic/Latino females aged 21 to 70 had a Pap test in the past 3 years, while the rate for the non-Hispanic Whites was only 72.8%. Similar findings were noted for mammograms among female HC patients aged 40 and over, with lower rate in the non-Hispanic White group (64.2%) than the sample average (70.3%). There were also differences among racial/ethnic groups for colorectal cancer screening, though the results were not statistically significant.

### Racial/ethnic differences in the receipt of pap tests

Table 2 summarizes the odds ratios (ORs) and 95% confidence intervals (CI) for factors predictive of receiving a Pap test in the past 3 years for women 21–70 years of age. Unadjusted models for Pap tests showed minorities had higher odds than that of non-Hispanic Whites. After adjusting for potential confounders, statistically significant racial differences were found between non-Hispanic Whites and African Americans. African Americans had higher odds than Whites (OR: 1.92, 95% CI: 1.44–2.55, *p* < 0.001) of receiving Pap tests. Similar findings were noted between non-Hispanic Whites and Hispanic/Latinos, with higher odds among Hispanic/Latinos than Whites (OR: 1.53, 95% CI: 1.09–2.15, *p* < 0.05). Elderly, U.S. born respondents were less likely to participate in a Pap test (OR: 0.95, 95% CI: 0.94–0.96, *p* < 0.001, OR: 0.52, 95% CI: 0.37–0.72, *p* < 0.001, respectively). Patients who were currently insured were more likely to receive a Pap test (OR: 1.68, 95% CI: 1.32–2.14, *p* < 0.001).

**Table 2:** Odds ratios and 95% confidence intervals for factors predicting receipt of Pap test in past 3 years for women 21 to 70 years: 2014 health center patient

	(*n* = 3168)		
	Unadjusted OR	Adjusted OR	(95% CI)
Race/Ethnicity (NH White)	Reference	Reference	
Hispanic/Latino	2.64****	1.53*	**(1.09, 2.15)**
NH African American	2.03****	1.92****	**(1.44, 2.55)**
NH “other”	1.83****	1.32	(0.95, 1.85)
Age		0.95****	**(0.94, 0.96)**
High school graduate		0.91	(0.73, 1.15)
U.S. born		0.52****	**(0.37, 0.72)**
Body mass index (0–24.99)		Reference	
Overweight (25.0–29.99)		0.67*	**(0.49, 0.92)**
Obese (30.0 or higher)		0.73*	**(0.55, 0.98)**
Current smoker		0.84	(0.66, 1.06)
Currently insured		1.68****	**(1.32, 2.14)**
Has a usual source of care		2.14	(0.93, 4.90)
Has Cancer		1.36	(0.93, 2.00)

*Significant at *p* < 0.05, ****Significant at *p* < 0.001

### Racial/ethnic differences in the receipt of mammograms

Table 3 shows the odds ratios and 95% confidence intervals for factors predictive of receiving a mammogram test in the past 2 years for women aged 40 and older. In the unadjusted model, race/ethnicity was found to be significantly associated with having a mammogram test. Whites continued to have lower odds of receiving breast cancer screening than the other three minority groups (*p* < 0.001). After additionally adjusting for other potential confounders, racial/ethnic differences were still statistically significant between African Americans (OR = 1.96, 95% CI: 1.46–2.64, *P* < 0.001) and non-Hispanic Whites, as well as between the other groups and non-Hispanic Whites (OR = 1.46, 95% CI: 1.03–2.07, *P* < 0.05). Significant differences were also observed for tobacco use, health insurance status, and being U.S. born. Patients who were U.S. born and current smokers were less likely to participate in a mammogram test (OR = 0.57, 95% CI: 0.41–0.79, *P* < 0.001 and OR = 0.56, 95% CI: 0.44–0.73, *P* < 0.001, respectively), while being currently insured was associated with higher odds of receiving mammogram test (OR = 1.79, 95% CI: 1.38–2.32, *P* < 0.001).

**Table 3:** Odds ratios and 95% confidence intervals for factors predicting mammogram receipt in the past 2 years for women 40 years and older: 2014 health center patient

	(*n* = 1942)		
	Unadjusted OR	Adjusted OR	(95% CI)
Race/Ethnicity (NH White)	Reference	Reference	
Hispanic/Latino	2.13****	1.34	(0.94, 1.92)
NH African American	2.02****	1.96****	**(1.46, 2.64)**
NH “other”	2.26****	1.46*	**(1.03, 2.07)**
Age		0.99	(0.98, 1.00)
High school graduate		1.03	(0.82, 1.30)
U.S. born		0.57***	**(0.41, 0.79)**
Body mass index (0–24.99)		Reference	
Overweight (25.0–29.99)		0.94	(0.69, 1.28)
Obese (30.0 or higher)		1.00	(0.75, 1.32)
Current smoker		0.56****	**(0.44, 0.73)**
Currently insured		1.79****	**(1.38, 2.32)**
Has a usual source of care		1.18	(0.38, 3.62)
Has Cancer		1.07	(0.76, 1.50)

*Significant at *p* < 0.05, ***Significant at *p* < 0.005, ****Significant at *p* < 0.001

### Racial/ethnic differences in the receipt of colorectal cancer screenings

Table 4 shows the odds ratios and 95% confidence intervals for factors predictive of receiving a colorectal cancer screening (endoscopy or fecal occult blood tests) among adults aged 50 and older. In the unadjusted model, African Americans and others were more likely to receive the tests compared with non-Hispanic Whites. However, the differences between African Americans and non-Hispanic Whites were insignificant. After additionally adjusting for potential confounders, racial/ethnic disparities were found between African Americans and non-Hispanic Whites (OR = 1.28, 95% CI: 1.02–1.60, *p* < 0.05). Regarding other factors associated with the receipt of endoscopy or fecal occult blood tests, significant differences were found among seven of nine measures in the adjusted model. The elderly were more likely to receive the test (OR = 1.05, 95% CI: 1.03–1.06, *P* < 0.001). Respondents with high school degrees had higher odds of receiving an endoscopy or fecal occult blood test (OR = 1.26, 95% CI: 1.06–1.51, *P* < 0.001). The adjusted odds were also higher among overweight patients (OR = 1.31, 95% CI: 1.05–1.63, *P* < 0.05) and obese patients (OR = 1.42, 95% CI: 1.15–1.75, *P* < 0.001) than patients with normal BMI. Current smokers were less likely to have a colorectal screening (OR = 0.72, 95% CI: 0.6–0.87, *P* < 0.001). Moreover, patients who currently have health insurance (OR = 1.91, 95% CI: 1.54–2.35, *P* < 0.001), a usual source of care (OR = 2.92, 95% CI: 1.41–6.04, *P* < 0.001) and cancer history (OR = 1.80, 95% CI: 1.32–2.46, *P* < 0.001) reported higher odds of receiving the tests.

**Table 4:** Odds ratios and 95% confidence intervals for factors predicting current receipt of lower endoscopy^a^ or fecal occult blood test: 2014 health center patient

	(*n* = 2653)		
	Unadjusted OR	Adjusted OR	(95% CI)
Race/Ethnicity (NH White)	Reference	Reference	
Hispanic/Latino	0.88	0.92	(0.70, 1.22)
NH African American	1.07	1.28*	**(1.02, 1.60)**
NH “other”	1.30*	1.26	(0.96, 1.66)
Age		1.05****	**(1.03, 1.06)**
Female		1.08	(0.91, 1.28)
High school graduate		1.26**	**(1.06, 1.51)**
U.S. born		0.91	(0.71, 1.16)
Body mass index (0–24.99)		Reference	
Overweight (25.0–29.99)		1.31*	**(1.05, 1.63)**
Obese (30.0 or higher)		1.42***	**(1.15, 1.75)**
Current smoker		0.72***	**(0.60, 0.87)**
Currently insured		1.91****	**(1.54, 2.35)**
Has a usual source of care		2.92***	**(1.41, 6.04)**
Has Cancer		1.80****	**(1.32, 2.46)**

*Significant at *p* < 0.05, **Significant at *p* < 0.01, ***Significant at *p* < 0.005, ****Significant at *p* < 0.001

^a^Sigmoidoscopy or colonoscopy

## Discussion

The current study presents nationally representative estimates of the receipt of three types of cancer screening services among patients in U.S. federally qualified health centers. Overall, the rates of receiving three types of cancer screening (Pap test, mammogram, colorectal cancer screening) were comparable and even higher among HC patients than the rates among the U.S. general population [16], though the HC patients were socioeconomically more vulnerable and may have constrained choices of health care services [21]. Specifically, the comparable rates of receiving Pap test, mammogram, colorectal cancer screening between HC patients and national general population were 81.9% vs. 81.4, 70.3% vs. 65.9, and 60.2% vs. 57.2% [2, 16]. Despite evidence about the benefits of cancer screening and its improvements for patient survival, challenges remain among the low-income population, largely due to disparities affecting those who are less educated, uninsured, and living below poverty level. However, the current results suggest that as a key component of the health care safety-net, HCs have successfully provided preventive care services for medically underserved populations, many of whom have increased risk of developing cancer [24]. Around one in twelve persons in the U.S., including one in five uninsured, one in six rural residents, one in ten children, and more than 330,000 veterans, are served by HCs [24]. For HC enrollees, Medicare waives Part B coinsurance and deductibles for the USPSTF-recommended grade A or B preventive services [5]. HCs have provided access to essential preventive services and timely care critical for healthy residents, people with higher levels of health risks, cancer patients, and survivors with cancer [9].

Both bivariate and multivariate results showed there were racial/ethnic differences in the likelihood of receiving cancer screening services among HC patients. However, the differences did not favor non-Hispanic Whites. The results were especially notable in two measures for women’s cancer prevention. Non-Hispanic Blacks were more likely than non-Hispanic Whites to receive Pap tests as well as mammograms. While analyses of national data had revealed that non-Hispanic Whites had the highest rates of receiving Pap tests, mammograms, and colorectal cancer screenings [2], our HC data showed that disparities between non-Hispanic Whites and each of the three minority groups were not in favor of non-Hispanic Whites. Though our results showed that non-Hispanic whites were more likely to have been diagnosed with cancer in the past, we controlled this covariate in our multivariate models, and the multivariate results showed reverse racial/ethnic disparities still existed after additionally adjusting for potential confounders. These results imply that reverse disparities between whites and the other groups in cancer screening, which may be due to many HC-specific initiatives, such as coverage expansions, have helped reduce longstanding disparities in preventive health care for racial minorities and low income population [14, 21]. Moreover, there have been many significant national and regional programs and policies that improve cancer screening for vulnerable populations such as those served by HCs. For example, HRSA announced a supplemental funding opportunity to improve cervical cancer screening by supporting HCs in achieving Patient-Centered Medical Home transformation [15]. Regarding colorectal cancer screening, Primary Care Associations (PCAs) were state or regional nonprofit organizations that committed to supporting colorectal cancer screening efforts by providing training and technical assistance to safety-net providers [8]. HCs have also worked towards reducing disparities in accessing breast cancer screening using several strategies, such as providing mammography services directly to underserved populations and referring HC patients to other facilities [18].

The status of being U.S. born was found to be associated with two types of cancer prevention measures. Those who were born outside the U.S. were associated with higher odds of receiving breast cancer screenings and cervical cancer screenings, which may be one possible reason for the disadvantages in cancer screening among non-Hispanic Whites. Most non-Hispanic Whites were originally from the U.S., compared with other racial/ethnic groups [17]. Insured patients were found to be more likely than the uninsured to receive the three cancer screening services, which was consistent with a previous study [19] and national findings [30]. Disparities among uninsured HC patients may imply that socioeconomic status, such as nationality and insurance status, may be seen as co-determinants of disparities in health care. Additional policy and infrastructure support are required to tackle both socioeconomic and race-based health disparities.

There were several limitations with this study. First, due to the cross-sectional nature of the survey data, we cannot make causal inferences from the findings. Second, although our analyses accounted for various potential confounding factors, we may have overlooked other factors related to the outcome indicators due to the limitation of secondary data. Third, the data were collected through self-reported surveys, which may be subject to recall or response biases. Because of the long period for colorectal cancer screening measure (e.g., colonoscopy/sigmoidoscopy screening in the past 10 years), there is a good chance that patients could have received the services at a time when they were not health center patients. Moreover, the included patients in this survey were those who had visited a HC at least once in the past year. This aspect of the study may have excluded the most vulnerable and underserved patients. Future studies may use quasi-experiment design and include this subgroup to make more rigorous estimations.

One strength of the study was its representativeness by using U.S. nationwide patient-level data of HC patients. Our study is the most recent national study to assess the differences in receipt of cancer preventive services among patients seen by HCs, who are a subpopulation of the nation’s most vulnerable and underserved populations. We found that this group of patients is able to receive a comparable level of cancer prevention compared with the general American population. Moreover, our findings suggest that federally qualified health centers play an integral role in providing equitable access to preventive services. Therefore, HCs are uniquely positioned to lead efforts to provide opportunities for reducing mortality and morbidity among HC populations by ensuring early screening and detection among this vulnerable segment of the population.

Important policy implications from this study indicate that as a key component of the health care safety-net, HCs continue to play a pivotal role in reducing health disparities among racial/ethnic subgroups. Since 2014, more and more previously uninsured HC patients have been gaining health insurance coverage and will be able to seek preventive cancer services through Medicaid expansion [22]. Thus, the ongoing repositioning by HCs to serve a vulnerable population with significant health needs seems likely to continue. Besides increasing their capabilities to provide treatment, HCs may focus on building strong health risk interventions, focusing on cancer-associated risk factors, and controlling cancer prevalence among the vulnerable segment of the population, which may in turn help reduce health care costs in the long term and improve the role of HCs in promoting community-based health care. Moreover, there is also much room for improvement on cancer screening rates, including improving the rates for non-Hispanic whites. In addition to ongoing policies and initiatives, new initiatives should emphasize collaboration, teamwork, and community-oriented recourses, as well as cancer prediction and decision-making support to further assist patients’ self-management and health maintenance.

## Data Availability

The data that support the findings of this study are available from the Bureau of Primary Health Care, Health Resources and Services Administration (HRSA).
